# Varicella Pneumonia

**DOI:** 10.5334/jbsr.1594

**Published:** 2018-08-13

**Authors:** Laurent Médart, Jeremy Liners, Jean-Baptiste Duquenne

**Affiliations:** 1 CHR Liège, BE

**Keywords:** VZV Pneumonia, immunocompetent adult infection

## Report

**Figure 1:**
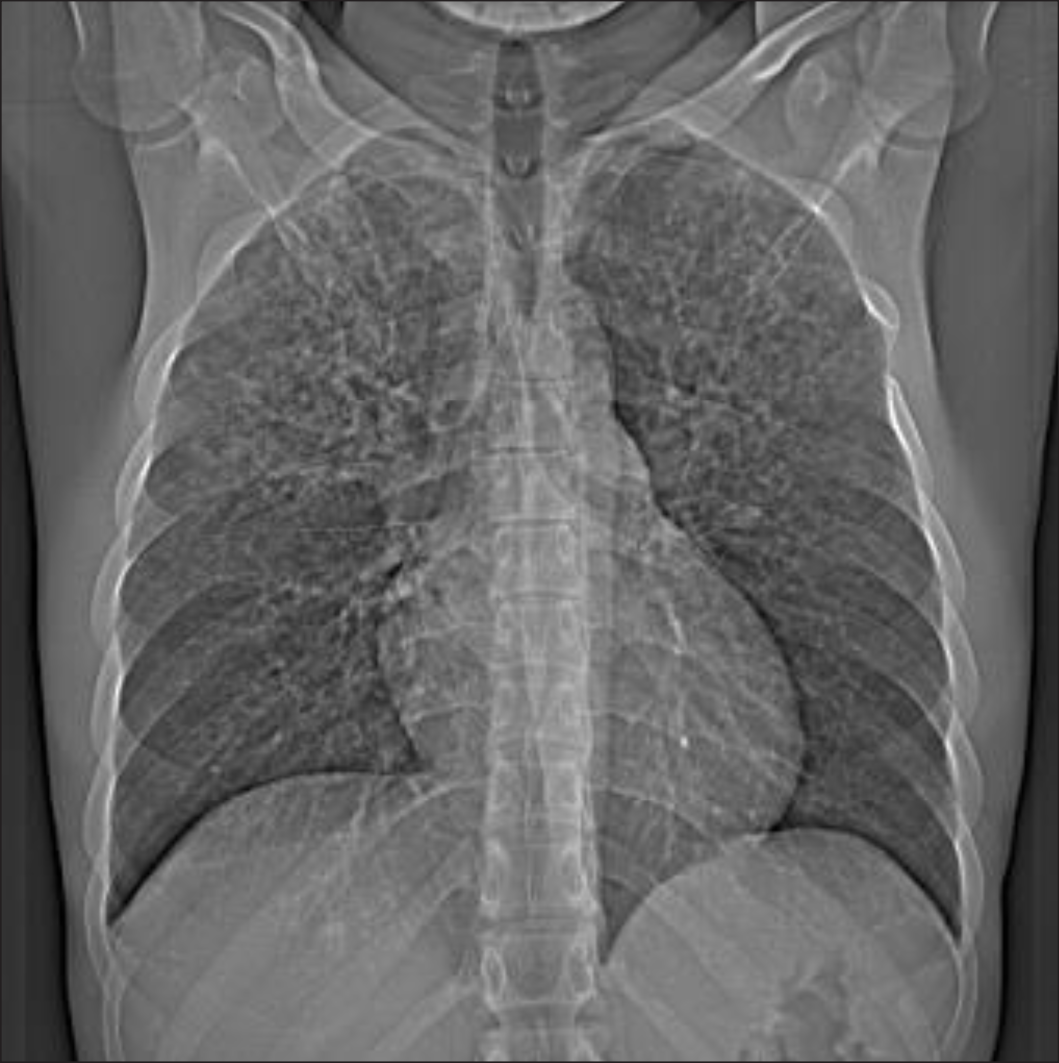


**Figure 2:**
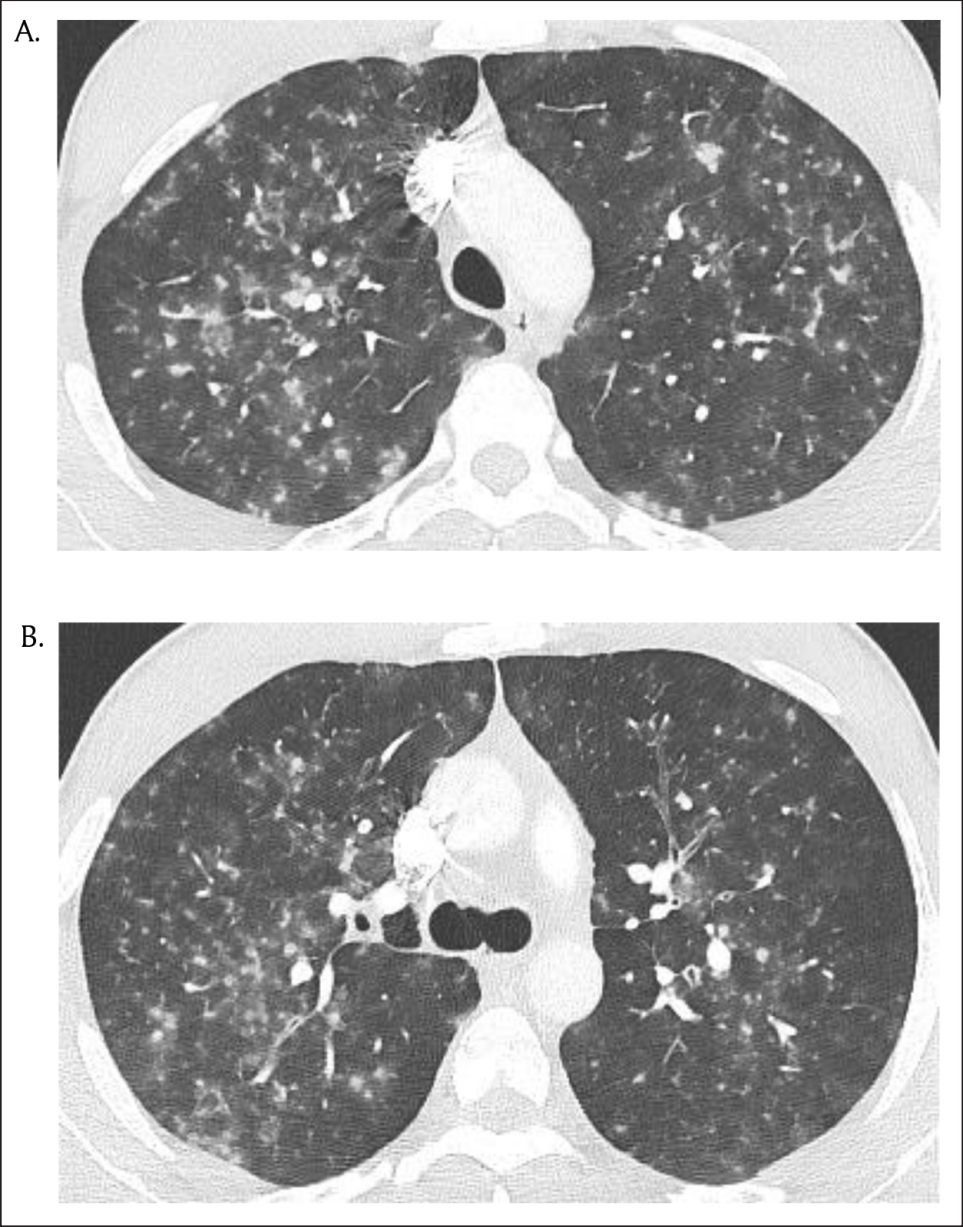


A 25-year-old man presented to the emergency room for slight hemoptysis. He was diagnosed with chickenpox three days prior, after contamination by his three-year-old daughter. His family doctor started a treatment consisting of Atarax 25 mg and oral Acyclovir 200 mg five times a day. Clinical presentation included painful cutaneous rash, cough with bloody filaments, chills and absence of dyspnea. Biological presentation includes slight elevation of the C-reactive protein and D-dimer levels. Chest Computed Tomography (CT) revealed multi-focal poorly defined centrilobular nodules and micronodules with peripheral ground glass and upper lobe predominance (Figures 1, 2A and 2B). The patient recovered from chickenpox 10 days later and avoided Intensive Care Unit (ICU) admission. Control CT at one month revealed persistence of some nodules and profusion of dense micronodules with total clearance of ground glass opacities.

## Discussion

Varicella Pneumonia is an uncommon complication of chickenpox, more frequent in older teenagers and adults than in children. This complication harbors significant morbidity and mortality for a vaccine-preventable disease. The proportion of adults susceptible for chickenpox is around 7% in economically-advanced nations. Among those undergoing chickenpox, some form of pulmonary illness will be noticed in 5–15%. Risk factors for varicella pneumonia include immunosuppression, pregnancy, heavy smoking, old age, COPD and severe cutaneous rash [1].

The clinical diagnosis is made on the typical skin involvement. Varicella pneumonia induce tachypnea, chest tightness, cough, dyspnea, fever, pleuritic chest pain or hemoptysis. Chest symptoms may start before the skin rash. The risk of developing respiratory failure requiring artificial ventilation is difficult to predict.

Varicella infection may involve other organs in addition to skin: lungs, brain, eyes, bone marrow, kidneys and liver.

Viral pneumonias in adults are due to numerous pathogens, the radiographic and CT findings are variable and overlapping. Varicella pneumonia findings on chest radiography and CT are not pathognomonic but differ slightly from other viral infections.

Chest radiography and HRCT imaging are useful in acute and past varicella pneumonia. The former is characterized by multiple 5- to 10-mm ill-defined nodules with surrounding ground-glass; often nodules may be confluent and present in different areas of the lungs. The latter is characterized by profusion of completely calcified micronodules in a miliary distribution.

Standard therapy is 7 to 10 days of Acyclovir, oral or IV therapy depending on severity. Adjunctive steroid therapy is controversial but needs to be discussed in severe infections. Nearly 40% of patients with VZV pneumonia require mechanical ventilation. In case of fulminant or refractory respiratory failure, ECMO (extracorporeal membrane oxygenation) should be an option.

Mortality rate of varicella pneumonia is improving nowadays (6% according to recent data). This is likely due to early diagnosis, treatment by Acyclovir and modern ICUs.
